# Common mental disorders and its associated factors and mental health care services for Ethiopian labour migrants returned from Middle East countries in Addis Ababa, Ethiopia

**DOI:** 10.1186/s12913-020-05502-0

**Published:** 2020-07-23

**Authors:** Melkie Tilahun, Abdulhalik Workicho, Dessie Abebaw Angaw

**Affiliations:** 1 Department of Epidemiology, Institute of health, faculty of public health, University of Jimma, Jimma, Ethiopia; 2 Department of Epidemiology and biostatistics, institute of public health, college of medicine and health science, University of Gondar, Gondar, Ethiopia

**Keywords:** Common mental disorder, migrants, Ethiopia

## Abstract

**Background:**

The migration of young Ethiopian men and women to the Middle East countries was mainly for economic reasons. The migration was largely irregular that posed a wide range of unfavorable life conditions for some of the migrants. The overall objective is to assess common mental disorders and its associated factors for Ethiopian migrants returned from the Middle East countries and to describe mental health care services targeting these migrants.

**Methods:**

The study employed a mixed-methods approach. For the quantitative part, a systematic random sampling technique was used to select a sample of 517 returnees. An interviewer-administered questionnaire based on Self Report Questionnaire-20 was used to collect data from respondents. The qualitative study employed a phenomenological study design to describe mental health care services. Key informant interviews and non-participant observation techniques were used to collect qualitative data.

**Results:**

The prevalence of common mental disorder among Ethiopian migrants returned from the Middle East countries was found to be 29.2%. education (AOR=2.90 95%CI: 1.21, 6.94), physical abuse (AOR=12.17 95%CI: 5.87, 25.22), not getting salary properly and timely (AOR=3.35 95%CI: 1.47, 7.63), history of mental illness in the family (AOR=6.75 95%CI: 1.03, 43.95), detention (AOR=4.74 95%CI: 2.60, 8.62), guilty feeling for not fulfilling goal (AOR=9.58 95%CI: 4.43, 20.71), and denial of access to health care (AOR=3.20 95%CI:1.53, 6.67) were significantly associated with a common mental disorder. Shelter based and hospital-based mental health care services were rendered for a few return migrants with mental disorders. The services were primarily targeted, female return migrants.

**Conclusion:**

The prevalence of common mental disorder was high among migrants returned from the Middle East countries. Despite the high burden of mental distress, only a small proportion of return migrants with mental illness is getting mental health care services.

## Background

People have migrated from one place to another since the start of human existence [1]. Even though human migration is not a new phenomenon, it has changed significantly in number and nature with the growth of globalization, including the ease of international transport and communication, the push and pull factors of shifting capital, effects of climate change, and periodic political upheaval, including armed conflict [2]. Migration has been increasing largely at the international level especially since the last decade [3]. Between 1990 and 2017, the number of international migrants worldwide rose by over 105 million, or by 69 percent. Globally, there were an estimated 258 million international migrants in 2017 [4]. Migration report estimates that if migration continues to increase at the same pace as in the last 20 years, the number of international migrants worldwide could be as high as 405 million by 2050 [5]. Africa is often seen as a continent on the move, with people escaping poverty, environmental disaster, or violent conflict. About 31 million Africans, or little more than 3 percent of the continent’s population, have migrated internationally [6].

There is evidence that outward migration has increased in Ethiopia in recent years [7]. Ethiopians leave their country either as regular or irregular migrants. Regular migration is defined by the International Organization for Migration (IOM) as "migration that occurs through recognized, authorized channels". IOM also defines irregular migration as "movement that takes place outside the regulatory norms of the sending, transit and receiving countries". Data from the Ministry of Labour and Social Affairs (MoLSA) indicate that approximately 460,000 Ethiopians migrated legally from their country between 2008 and 2013, mostly to the Middle East with the majority going to Saudi Arabia (79%), Kuwait (20%) and others to UAE and other countries [8]. The exact number of Ethiopian migrants to the Middle East is unknown as two-thirds of them migrate through irregular means [9]. It is estimated that up to 500,000 Ethiopian women are migrating to the Middle East for domestic work annually [10]. Ethiopian migrants to the Middle East are driven to migrate primarily for economic reasons [11]. The process of migration can lead to a whole spectrum of physical and mental health disorders [12]. There are numerous reports that many migrants are victims of fraud, forced labour, and physical, sexual, and psychological abuse by their employers or by traffickers, and a significant number develop psychological problems [7]. Many Ethiopian women working in domestic service in the Middle East face severe abuses, including physical and sexual assault, denial of salary, sleep deprivation, withholding of passports, confinement, and even murder [13].

Similar with the outward migration, return migration to Ethiopia has increased in the past decade [10]. Ethiopians return to their country due to various reasons such as to reunite with family or friends, investment, or repatriation and deportation [8]. The recent deportation of 170,000 Ethiopian migrants from the Kingdom of Saudi Arabia is one example of large-scale return migration in the country [14]. The deportation of the above-undocumented migrants was accompanied by severe human rights abuses, including arbitrary detention, theft of migrants' belongings, rape, beatings, and killings' that traumatized many of those who returned to Ethiopia [7]. Return migrants may have exceptional and increased mental health needs resulting from painful or traumatic experiences that they might face during the process of migration and/or during their stay in the country of destination [15]. A growing number of shreds of evidence show that Ethiopian returnees from different Middle East countries often have a variety of psychological disorders as they experience diverse problems at the various stages of their migration [16]. This, in turn, makes mental health to be a serious concern among Ethiopian migrants returning from the Middle East countries [9].

Studies suggest that mental health care and rehabilitation services are highly needed to be expanded to return migrants in Ethiopia [17]. However, return migration and mental health has received far too little attention in policy and crisis-intervention programs despite a large number of Ethiopian migrants with various mental health issues are returning from the Middle East countries. The National Mental Health Strategy which integrates mental health into primary health care systems to provide comprehensive, accessible and affordable mental health care for the public does not specifically address the special mental health care need of return migrants [18].

Even though hazards to general health and specifically to mental health rank among the top experiences of trafficking and migrant returnees, the services addressing health specifically mental health needs of returnees during the recovery phase of the migration process are sparse [9, 16, 17]. Therefore, this research aimed at studying the magnitude of common mental disorders and associated factors and mental health care practice that targets Ethiopian migrants returning in large number from Middle East countries.

### Conceptual framework

**Fig. 1:**
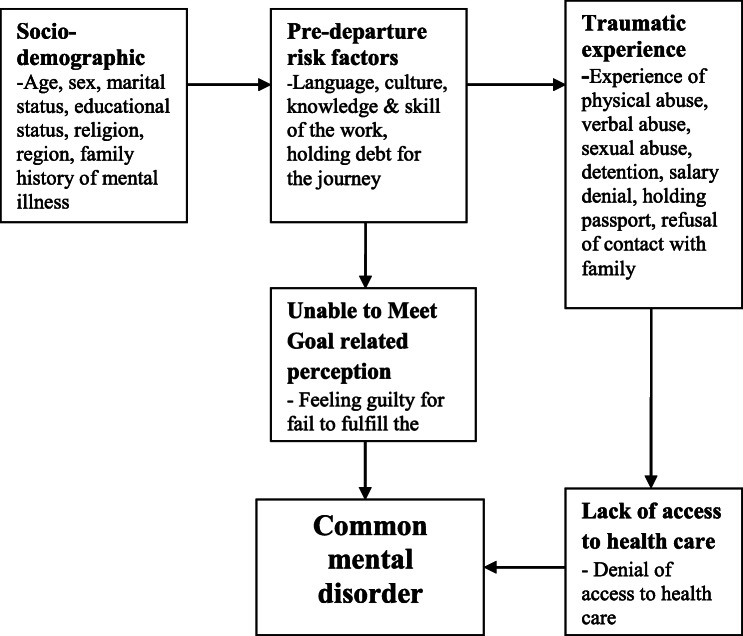
Conceptual framework of the study

The constructs of this conceptual framework were extracted from a bulk of literature written in the area. The framework depicts CMD or mental distress in return migrants is a result of socio-demographic characteristics, pre-departure risk factors, traumatic life experiences, goal-related perception, and access to health care in the destination country (Fig. 1).

## Methods

### Study design and setting

The study employed a mixed-methods research approach. The cross-sectional study design was used in the survey to assess the prevalence of common mental disorder and its determinants among Ethiopian migrants returning from the Middle East countries. The qualitative research applied a phenomenological study design to describe the dimension of mental health care services, opportunities, and challenges that were aimed at providing mental health care services for return migrants.

The study was conducted in Addis Ababa city which was the main gate to Ethiopian returnees from different parts of the world including those from the Middle East region via Bole International Airport. It is the hub for various actors working on rehabilitation and reintegration of return migrants. The main organizations working on rehabilitation and reintegration of return migrants in Addis Ababa include Addis Ababa City Administration Social and Labour Affairs Bureau, International Organization for Migration, Agar Ethiopia, Good Samaritan Association, Nolawi Services and St. Amanuel Mental Specialized Hospital. The study was conducted in the period between November 10, 2017, and December 21, 2017.

### Population

The population for the quantitative study was Ethiopian labour migrants returning from Middle East countries who were staying in the provisional center in Addis Ababa and who was not unconscious and not critically ill. The population of the qualitative study was staff of Addis Ababa City Administration Labour and Social Affairs Bureau, Agar Ethiopia, Good Samaritan Association, Nolawi Services, St. Amanuel Specialized Mental Hospital and Ministry of Health which specifically work on mental healthcare of return migrants from the Middle East countries.

### Sample size and sampling technique

The sample size was calculated using single population proportion formula with the prevalence rate of common mental disorder for the population (27.6%), which was based on a recent study conducted by Habtamu, Minaye, and Zeleke in 2017 [16]*; d* is the margin of error to be tolerated (4%) and $$ {Z}_{1-\raisebox{1ex}{$\alpha $}\!\left/ \!\raisebox{-1ex}{$2$}\right.} $$ is the reliability factor corresponding to the confidence level of 95% (1.96)$$ n=\frac{{Z_{1-\raisebox{1ex}{$\alpha $}\!\left/ \!\raisebox{-1ex}{$2$}\right.}}^2P\left(1-P\right)}{d^2} $$

The computed sample size was 480 and with a 10% non-response rate, the final sample size became **528**. A systematic random sampling technique was used to select samples from Ethiopian return migrants. A list of 1059 return migrants who arrived during the study period was obtained from a register of the National Disaster Risk Management Commission, the government body that coordinated tasks in the provisional center at that time. The sampling interval (k) was calculated by dividing the number of returnees (1059) by the sample size (528). The calculated sampling interval was 2. A lottery was drawn between the first two names of return migrants from the register to randomly select the first respondent. Then every other migrant’s names from the register were selected to be sampled and interviewed.

For the qualitative part, a purposive sampling technique was employed to select participants and institutions. Participants and institutions with relevant and rich information were selected deliberately for the study. Information about relevant staff for the study was obtained by asking the leaders of each organization. Information saturation was used to determine the point at which the data collection ends. Ten key informant interviews were conducted with staff of Addis Ababa City Administration Labour and Social Affairs Bureau, Agar Ethiopia, Good Samaritan Association, Nolawi Services, St. Amanuel Specialized Mental Hospital and Ministry of Health. The key informants were focal persons from each organization who had relevant and rich information about the matter. In the same way, three observations were conducted at Agar Ethiopia, Good Samaratian Association, and St. Amanuel Specialized Mental Hospital.

### Study variables and data collection procedure

Our dependent variable was a common mental disorder with the presence of 8 or more symptoms of mental distress in return migrants out of 20 symptoms in the SRQ-20. Socio-demographic departure, the experience of a traumatic event, health care, and goal-related perceptions were assessed quantitatively. The qualitative study explored the depth and dimensions of mental health care services available. It also explored opportunities and challenges the actors encounter. Contents that were related to mental health care services, opportunities, and challenges were identified.

A face to face interview was used to collect data from respondents in the quantitative study. A structured questionnaire based on the WHO Self Report Questionnaire-20 (SRQ 20) was used to assess mental distress among sample respondents in the past 30 days. The questionnaire was translated into the Amharic language. Selected participants were asked for their willingness to participate in the study. Data was collected from migrants who fulfilled the inclusion criteria. The inclusion criteria for the study were: who was not unconscious, who were not critically ill, and who were not below 18 years old. Three data collectors with a bachelor's degree in psychology collected data from respondents.

Two different data collection techniques were used to collect qualitative data. These were key informant interviews and observation. The KI participants were mental health practitioners who had rich experience in providing mental health services for the return migrants’ Key informant interviews were conducted to collect rich information about mental health care services from highly relevant staffs in those organizations which provide rehabilitative and mental health care services for return migrants. The key informant interview process was guided by a key informant interview guide. The key informant interviews were tape-recorded to capture all information given by the interviewees. After completion of each key informant interview, the facilitators thanked each participant for his/her willingness and time. A non-participant observation technique was used for the observation. The observation aimed to gather first-hand information on mental health services by observing sites where actual service is rendered. The first-hand information gathered through observation was the general setting of the service provider site, types of mental health services provided at the site, availability of adequate spaces for each service, availability of required materials and facilities for each service, appearance, and condition of mental health care clients, and interaction between service providers and clients. A structured observation checklist was used to guide the observation. Field notes were taken immediately after each observation. The investigator gathered the qualitative data from study participants and institutions

### Data quality management

For quantitative data quality, a standard WHO questionnaire with acceptable validity and reliability was used to collect data from respondents. The data collectors were trained for two days on data collection tools and data collection procedures. A filled questionnaire was checked for completeness and consistency by the investigator during data collection. Data entry was done in EpiData software to minimize data entry errors.

To enhance the trustworthiness of qualitative research, the triangulation of data collection methods was used to elicit information about the same issue using key informant interviews and observation. The data from the two methods were triangulated during interpretation. The researcher kept records on the process of conducting the research as it was undertaken for the audit trail at a later time to review different aspects of the research. The extensive description of the setting and participants of the study were provided. Besides, a detailed description of the findings with adequate pieces of evidence was provided in the form of quotes from participants’ interviews.

### Data analysis procedures

Quantitatively collected data were entered into EpiData version 3.1. The data were edited and cleaned carefully. Then, the dataset was exported to SPSS Version 20 for analysis. Descriptive statistics were run to summarize the background characteristics of the respondents, to determine the prevalence of CMD, and to examine the distribution of specific symptoms contained in SRQ-20 among the respondents. Logistic regression models were fitted to identify factors associated with CMD. Analyses of associations for CMD focused on the presence and absence of the disorder taking the score of eight and above as a cut of a point on the score of SRQ-20. The screening criterion for variables to be included in the multivariable regression was the P-value <0.25 in the bivariate regression model. The level of significance of association in the logistic regression model was determined at P-value <0.05.

For qualitative data, tape-recorded key informant interviews were firstly transcribed in Amharic and then translated into English. The transcribed notes were edited, formatted, and saved as a text file. Then, the transcript notes saved in a text file were imported into OpenCode version 4 software. Codes were assigned to segments of the text. Then after the codes were categorized into four themes. The field notes taken during observations were summarized, categorized, and analyzed based on the site of observation. Finally, the findings from the two data collection techniques were triangulated.

## Result

### Quantitative Results

#### Socio-demographic Characteristics

A total of 517 return migrants participated in the cross-sectional survey with a response rate of 97.9%. More than half (56.9 %) of the respondents were female. The mean age of the respondents was 27.5 (SD = +5.0) years. Regarding their religion, over half (56.3%) of them were Muslim followed by Orthodox (30.2%). In terms of respondents' education, 13.0% of them did not have formal education and 37.9% attended primary education. Most of the respondents were from Amhara (33.3%), Oromia (27.7%), and Tigray (22.4%) regions of Ethiopia where a little more than half (53.2%) of the return migrants were from rural areas of these regions (Table 1).

**Table 1:** Socio-demographic Characteristics of study participants

Characteristics	Number	Percent
Gender		
Female	294	56.9%
Male	223	43.1%
Total	517	100%
Age (years) mean±(SD)	27.5 +5.0	
Religion		
Orthodox	156	30.2%
Protestant	58	11.2%
Catholic	12	2.3%
Muslim	291	56.3%
Total	517	100%
Marital Status		
Single	261	50.5%
Married	245	47.4%
Separated/Divorced	11	2.1%
Ethnicity		
Amhara	163	31.5%
Oromo	146	28.2%
Tigre	119	23.0%
Gurage	60	11.6%
Others	29	5.6%
Education		
No formal education	67	13.0%
Grade 1-8	196	37.9%
Grade 9-10	155	30.0%
Grade 11 and above	99	19.1%
Region		
Amhara	172	33.3%
Oromia	143	27.7%
Tigray	116	22.4%
SNNPR	72	13.9%
Addis Ababa	11	2.1%
Others	3	0.6%
Residence		
Urban	242	46.8%
Rural	275	53.2%

#### Pre-departure Factors

The majority (86.5%) of the respondents did not know the language of the destination country before their migration. Only a few (11%) of them claimed that they had had the required skill for the type of work they supposed to do in the destination country. Regarding family pressure to migrate, the vast majority (89.2%) did not mention family pressure as a reason for their decision to migrate to the Middle East countries. Nearly half (48.2 %) of the respondents or their families took a loan to cover the cost of their journey to the Middle East country (Table 2).

**Table 2:** Pre-departure Characteristics of the study participants

Pre-departure Situation	Number	Percept (95% CI)
Knowledge of destination country’s language		
Yes	70	13.5% (95% CI= 10.6,16.6)
No	447	86.5% (95% CI=83.4, 89.4)
Knowledge of destination country’s culture		
Yes	71	13.7%(95% CI=10.6, 16.8)
No	446	86.3%(95% CI=83.2, 89.4)
Knowledge about the type of works supposed to do		
Yes	67	13.0%(95% CI=10.0, 16.4)
No	450	87.0%(95% CI=83.6,90.0)
Had the required skill for the work supposed to do		
Yes	57	11.0%(95% CI=8.3, 14.1)
No	460	89.0%(95% CI=85.9, 91.7)
Family pressure to migrate		
Yes	55	10.6%(95% CI=8.1, 13.2)
No	462	89.4%(95% CI=86.8, 91.9)
Loan for the journey		
Yes	249	48.2%(95% CI=44.1, 52.2)
No	268	51.8%(95% CI=47.8, 55.9)
Presence of mentally ill person in the family		
Yes	14	2.7%(95% CI=1.4, 4.3)
No	503	97.3%(95% CI=95.7, 98.6)

#### Experience of Traumatic Events and Access to Health Care

Nearly one-third (32%) of return migrants reported that they had experienced physical abuse during their journey or at the destination country in the Middle East. More than half (55.3%) of the returnees reported that they had encountered verbal abuse. Among the female returnees, 68 (23.1%) of them reported sexual abuse during their journey or at their stay in the destination country. Only one-fifth (20.7) of the respondents reported to get their salary timely and properly. Regarding confiscation of passport, 86.1% of the returnees reported their passport had been held forcefully by their employer. A third of the return migrants (32.9%) were refused to communicate their family through the telephone.

Among the total respondents, 36.4 % of them were detained either during their journey or at the destination country in the Middle East. More than sixty percent of the respondents reported that feeling guilty for unmet their primary goal of their migration. Slightly more than three-forth (76.8%) of the returnees were denied access to health care by their employer during their stay in the host Middle East country (Table 3).

**Table 3:** Traumatic Experience of study participants

Traumatic event	Number	Percept (95% CI)
Physical abuse		
Yes	165	31.9% (95% CI=27.8, 36.2)
No	352	68.1% (95% CI=63.8, 72.2)
Verbal abuse		
Yes	282	54.5% (95% CI=50.3, 58.8)
No	235	45.5% (95% CI=41.2, 49.7)
Sexual abuse (female only)		
Yes	68	23.1% (95% CI=18.8, 28.0)
No	226	76.9% (95% CI=72.0, 81.2)
Got salary timely		
Got salary timely and properly	107	20.7% (95% CI=17.4, 24.4)
Got salary sometimes	324	62.7% (95% CI=58.2, 66.2)
Never got salary	86	16.6% (95% CI=13.3, 20.3)
Employer hold passport forcefully		
Yes	445	86.1% (95% CI=83.4, 89.2)
No	72	13.9% (95% CI=10.8, 16.6)
Were refused to communicate/contact family		
Yes	170	32.9% (95% CI=28.8, 37.1)
No	347	67.1% (95% CI=62.9, 71.2)
Experienced detention during the journey and/or at destination country		
Yes	188	36.4% (95% CI=32.1, 40.6)
No	329	63.6% (95% CI=59.4, 67.9)
Guilty feeling for not fulfilling primary goal of migration		
Yes	318	61.5% (95% CI=57.6, 65.6)
No	199	38.5% (95% CI=34.4, 42.4)
Access to health care at destination country		
Yes	120	23.2% (95% CI=19.3, 26.7)
No	397	76.8% (95% CI=73.3, 80.7)

#### Prevalence of Common Mental Disorders

The prevalence of common mental disorder among Ethiopian migrants returned from the Middle East countries was found to be 29.2% (95% CI= 25.3, 33.3). The most frequent symptoms of common mental disorder reported by the respondents were: frequent headache (40.6%), feel unhappy (40.4%), nervousness (40.2%), bad sleep (39.7%), poor appetite (39.3%), feel tired all the time (35.4%) and easily tired (34.8%). Slightly more than a fifth (21.5%) of the respondents reported that they felt worthless in the past 30 days and suicidal ideation was reported by 17.4 % of the return migrants who participated in the study (Table 4).

**Table 4:** Symptoms of Common Mental Disorder Reported by the study participants

Symptom	Yes	No
Frequent Headache	210 (40.6%)	307 (59.4%)
Poor appetite	203 (39.3%)	314 (60.7%)
Bad sleep	205 (39.7%)	312 (60.3%)
Easily frightened	147 (28.4%)	370 (71.6%)
Hands shake	86 (16.6%)	431 (83.4%)
Nervousness, tensed or worried	208 (40.2%)	309 (59.8%)
Poor digestion	140 (27.1%)	377 (72.9%)
Trouble in thinking clearly	118 (22.8%)	399 (77.2%)
Feel unhappy	209 (40.4%)	308 (59.6%)
Cry more than usual	132 (25.5%)	385 (74.5%)
Difficult to enjoy daily activities	151 (29.2%)	366 (70.8%)
Difficult to make decisions	116 (22.4%)	401 (77.6%)
Suffering of daily work	99 (19.1%)	418 (80.9%)
Unable to play a useful part in life	121 (23.4%)	396 (76.6%)
Lost interest in things	147 (28.4%)	370 (71.6%)
Feel worthless	111 (21.5%)	406 (78.5%)
Thought of ending life	90 (17.4%)	427 (82.6%)
Feel tired all the time	183 (35.4%)	334 (64.6%)
Stomach ache	134 (25.9%)	383 (74.1%)
Easily tired	180 (34.8%)	337 (65.2%)

#### Factors Associated with Common Mental Disorder

In the multivariable logistic regression model; Education (AOR=2.90 95%CI: 1.21, 6.94), experience physical abuse (AOR=12.17 95%CI: 5.87, 25.22), not getting salary properly and timely (AOR=3.35 95%CI: 1.47, 7.63), history of mental illness in the family (AOR=6.75 95%CI: 1.03, 43.95), experience detention (AOR=4.74 95%CI: 2.60, 8.62), guilty feeling for not fulfilling goal (AOR=9.58 95%CI: 4.43, 20.71), and denial of access to health care in the destination country (AOR=3.20 95%CI:1.53, 6.67) were significantly associated with CMD (see table 5).

**Table 5:** Multivariable logistic regression model for factors associated with common mental disorder among Ethiopian labor migrants returned from the Middle East Countries between November 10, 2017 and December 21, 2017

Variable	CMD		COR	95% CI		AOR	95% CI	
	Yes N (%)	No N (%)						
**Gender**								
Female	95 (63%)	199 (54%)	.702	.476	1.036	.841	.461	1.535
Male	56 (37%)	167 (46%)	1.0			1.0		
**Marital status**								
Single	84 (55.6%)	177 (48.4%)	2.529	.750	8.521	3.327	.498	22.243
Married	61 (40.4%)	184 (50.3%)	3.620	1.067	12.281	5.322	.783	36.177
Separated/Divorced	6 (4.0%)	5 (1.4%)	1.0			1.0		
**Residence area**								
Urban	64 (42%)	178 (49%)	1.287	.878	1.886	.211	.080	.561
Rural	87 (58%)	188 (51%)	1.0			1.0		
**Education**								
Grade 1-8	59 (39.1%)	137 (37.4%)	2.254	1.277	3.977	2.900	1.211	6.945
Grade 9-10	42 (27.8%)	113 (30.9%)	2.611	1.439	4.738	10.505	3.121	35.358
Grade 11and above	17 (11.3%)	82 (22.4%)	4.682	2.305	9.510	15.309	3.786	61.893
No formal education	33 (21.9%)	34 (9.3%)	1.0			1.0		
**Presence of mentally ill person in family**								
Yes	2 (1%)	12 (3%)	2.525	.558	11.422	6.758	1.039	43.955
No	149 (99%)	354 (97%)	1.0			1.0		
**Physical abuse**								
No	47 (31.1%)	305 (83.3%)	11.064	7.120	17.191	12.174	5.876	25.224
Yes	104 (68.9%)	61 (16.7%)	1.0			1.0		
**Verbal abuse**								
No	36 (23.8%)	199 (53.5%)	3.807	2.483	5.835	.463	.218	.981
Yes	115 (76.2%)	167 (46.5%)	1.0			1.0		
**Sexual abuse**								
No	51 (53.7%)	175 (87.9%)	6.291	3.497	11.315	1.142	.393	3.318
Yes	44 (46.3%)	24 (12.1%)	1.0			1.0		
**Got salary timely**								
Got salary properly	20 (13.2%)	87 (23.8%)	5.242	2.750	9.995	.344	.164	.724
Got salary sometimes	84 (55.6%)	240 (65.6%)	3.443	2.105	5.631	3.354	1.474	7.631
Never got salary	47 (31.1%)	39 (10.7%)	1.0			1.0		
**Being refused to communicate family**								
yes	94 (62%)	253 (69%)	1.358	.913	2.019	.860	.464	1.596
No	57 (38%)	113 (31%)	1.0			1.0		
**Detention**								
yes	51 (33.8%)	276 (75.8%)	6.194	4.095	9.369	4.741	2.607	8.622
No	100 (66.2%)	88 (24.2%)	1.0			1.0		
**Feel guilty for not fulfilling goal**								
yes	12 (7.9%)	187 (50.4%)	12.101	6.482	22.592	9.582	4.433	20.714
No	139 (92.1%)	179 (49.6%)	1.0			1.0		
**Access to health care**								
Yes	23 (15.2%)	97 (26.5%)	2.007	1.216	3.312	3.203	1.537	6.674
No	128 (84.8%)	269 (73.5%)	1.0			1.0		

#### Qualitative Research Findings

A total of ten participants were interviewed in the qualitative interviews. Half of them were female. Four main themes were identified from the content analysis of qualitative research. These were (i) the mental health problems of return migrants through providers' eyes, (ii) mental health care services being rendered for return migrants (iii) the existing opportunities for mental health care providers, and (iv) the challenges encountered by mental health care providers. Direct quotes from transcripts are provided to illustrate these themes. Excerpts or quotations from interviews with participants are identified by a code corresponding to Table 6.

**Table 6:** Characteristics of key informant interview participants from mental health care service providing organizations between November 10, 2017 and December 21, 2017 Addis Ababa

Participant code	Sex	Organization	Position of participant
P1	Female	St.Amanuel Hospital/Good Samaritan Association	Prescriber
P2	Female	Good Samaritan Association	Executive Director
P3	Male	Agar Ethiopia	Executive Director
P4	Female	St.Amanuel Hospital/Agar Ethiopia	Psychiatric Nurse
P5	Male	St.Amanuel Hospital/Agar Ethiopia	Liaison Office Head
P6	Female	St.Amanuel Hospital	Psychiatric Nurse
P7	Female	St.Amanuel Hospital	Prescriber
P8	Male	AA Bureau of Labour & Social Affairs	Team Leader
P9	Male	Nolawi Services Ethiopia	Executive Director
P10	Male	Ministry of Health	Mental Health Officer

#### The Mental Health Problems of Return Migrants through Providers’ Eyes

The mental health problem of return migrants was vast and increasing from time to time. Participants agreed that female migrants were more affected by mental illness than their male counterparts. Those with mental health problems were brought to service providers in disturbing health conditions. The majority of return migrants were suffering from anxiety and depression and few of them were diagnosed with a severe form of mental illness like schizophrenia. Among the participants a female psychiatric nurse from Amanuel mental hospital who was also working in Agar Ethiopia supported the above idea by expressing:

*"In my understanding, almost all return migrants are affected by a mental health problem. The problem is immense and increasing from time to time. Most of the time, they are suffering from depression. They are also suffering from acute psychotic disorders. Few of them are suffering from a chronic psychotic disorder such as schizophrenia"* (P4).

Another female participant from Good Samaritan Association agreed with the aforementioned idea by describing:

*"Most of the returnees coming to us were disoriented and traumatized. Some of them were with physical injuries. Some were with bad odour from their mouths and blood in their urine… Few were tested positive for HIV and TB"*(P2).

Many Ethiopian labor migrants took an unsafe route in their migration to the Middle East countries.

Some of the participants claimed the Ethiopian government's ban of migration to the Gulf Arab countries exacerbated the illegal migration to the region. Most of the migrants were poorly prepared for the working and living conditions in the destination countries making them prone to abuse and mistreatment during their journey and at their destination. For most of the migrants, their work and stay in the employer house were full of exploitation and violation of their rights. The Kafala sponsorship system that practiced in the region also played a role in the exploitation and right violations of Ethiopian labour migrants in the Gulf Arab countries. A male participant working in Nolawi Services explained the poor preparation of Ethiopian labour migrants for the supposed domestic work and life in the destination country:

"*Ethiopian migrants do not have appropriate skills for the work they supposed to do. They do not have pre-departure orientation or training on the skill required for the work. They do not know the basic Arabic language. They are unaware of the culture of the destination country. They directly go to Arab countries without basic training and preparation. They are not aware of the working condition in the destination country*" (P9).

A female psychiatric prescriber from Amanuel hospital also working in Good Samartian Association described the above abuse and mistreatment the Ethiopian labour migrants faced:

"*Most of the time, the mistreatment and abuse are started from here in Ethiopia by traffickers. During their journey to the Arab countries, they face rape, torture, insult, and more. Once they arrive at the Arab country, the mistreatment and abuse are continued. They are not allowed to communicate with family and friends. They are starved. They are forced to drink unclean pipe water used for cleaning purposes"* (P1).

#### Mental Health Care Services Being Rendered for Return Migrants

Ethiopian return migrants with mental health problems got mental health care services at two settings namely at the rehabilitation center and a mental hospital. According to the participants and observations by the investigator, there were only three organizations that provided mental health care services for return migrants. Two of them were providing a rehabilitation center or shelter-based mental health services. The remaining one provided hospital-based mental health care.

#### Rehabilitation Center or Shelter Based Services

The rehabilitation center based mental health care services were provided by Agar Ethiopia and Good Samaritan Association. The two organizations were providing basic mental health care and rehabilitation services to returnees with mental illness. The services provided by the two organizations were shelter, food, hygienic materials, clothes, medical service through referral, psychological counseling, recreational therapy even though not well organized, reunification with family, life skill training, vocational skill training, and economic strengthening through linking with micro-finance institutes. The rehabilitation centers were aimed at providing mental health care services for female return migrants only. Few female migrants with severe mental illness got the services. Among the participants, a staff of Agar Ethiopia explained about the services his organization was providing:

"*We are providing a range of mental health care related services to return migrants with mental problems. The services we are providing to them are food, shelter, counseling, recreational therapy, life skill training, vocational skill training, reunification with family, and economic empowerment. We provide mental health care services for only female returnees at our rehabilitation center. Only a few of the returnees with mental problems are brought to our rehabilitation center as most of them do not show apparent signs of severe mental illness. The majority of returnees with mental illness are left on the street.*"(P3)

During observation of Agar Ethiopia's rehabilitation center, the investigator observed 8 return migrants with mental illness. All of them were female and one of them had a baby. The middle size premise of the center was neat and free from bad odor and hazardous objects at the time of observation. Similarly, while observing the Good Samaritan Association's rehabilitation center, the investigator observed 5 mentally ill return migrants. All of them were female. The investigator observed paintings made by mentally ill return migrants hanged on one side of the wall. The center was a one-floor building with a small size compound.

#### Hospital-Based Services

St Amanuel hospital provided outpatient and inpatient medical services for the general public with mental illness. The hospital rendered mental health care services for return migrants with mental illness who were mainly brought by Agar Ethiopia, Good Samaritan Association, and Ethiopian Airports Enterprise. These services were: psychiatric assessment, prescription of medication or biological therapy, psychological counseling, ward admission, and reunification with a family. A female psychiatric nurse of Amanuel hospital described the type of services the hospital rendered for return migrants:

"*Amanuel hospital is providing a range of mental health care services for return migrants with mental illness. The hospital provides psychiatric assessment, counseling services, prescription of medication, inpatient services through ward admission, outpatient services, and reunification with family*" (P6).

The observation in Amanuel hospital revealed that it was situated in one of the most bustling areas of the city. The investigator observed so many mentally ill male and female patients wearing a hospital gown. There were separate wards for male and female patients. The hospital compound was crowded with many outpatient and inpatient clients and their families/relatives. The waiting areas around examination rooms and registration/card rooms were full of patients and their families and relatives.

#### The Existing Opportunities for Mental Health Care Providers

There were limited opportunities for organizations working on mental health care of return migrants. The increased attention is given to mental health globally and nationally was considered as an opportunity for the expansion of mental health care services. Limited support provided by the government was helping to strengthen the capacity of the actors working on mental health care of return migrants. The presence of Amanuel mental hospital in Addis Ababa was an opportunity to diagnose and treat mentally ill return migrants brought by the two organizations working on mental health care of return migrants. Participants said that the dedication of staff and leaders in handling the challenging task of caring for mentally ill return migrants was an opportunity for organizations working on the area. Among the participants' Agar Ethiopia staff described the support his organization got from the government as worth mentioning opportunity:

"*The city government is supporting us in a limited way. For instance, AA BOLSA is working closely with us as it is part of their responsibilities. The Addis Ababa City Civil Society Agency has donated us a vehicle. Addis Ababa City Disaster Preparedness Bureau has granted us an emergency fund of one and a half million birr. Addis Ababa City Council is on the process of providing us 3 hectares of land for construction of rehabilitation center*" (P3).

A male staff of Amanuel hospital who was also serving as psychiatric staff in Agar Ethiopia explained the attention the Ethiopian government gave to mental health:

"*The government is working to improve mental health care services in the country. It trains a large number of mental health professionals. Also, it decentralizes mental health care services to different levels of government health facilities*" (P5).

#### The Challenges Encountered by Mental Health Care Providers

Mental health care providers shave encountered many challenges in their effort to deliver mental health care services to return migrants. Inadequate funding to expand services for all needy returnees was a major challenge for most of the providers. The limited capacity to expand mental health care services for all needy return migrants was another challenge of the actors in the area. Overcrowding and a shortage of beds in the hospital was a chronic problem for providing mental health care services for returnees and the general public. Almost all return migrants came to the hospital unaccompanied by family or relatives so that it posed a challenge to assess, treat, and follow them. Lack of awareness about mental illness and stigma against a mentally ill person was also one of the challenges that mental health care providers encountered. Lastly, the exaggerated need for economic support of return migrants recovered from mental illnesses posed a challenge for the actors struggling with meager resources. Agar Ethiopia staff alluded that lack of adequate space was a bottleneck for them to provide service for all needy return migrants:

"*We do not have enough space to provide mental health care service for all needy return migrants including male returnees*" (P3).

One of Amanuel hospital staff also described the aforementioned challenge as follow:

"*There is a shortage of bed to admit all needy return migrants with severe mental illness even though efforts to admit this group of the society are in place*" (P5).

## Discussion

The prevalence of CMD among Ethiopian labour migrants returned from the Middle East countries was found to be 29.2% (95% CI= 25.3, 33.3). A similar study reported a prevalence rate of CMD to be 27.6 % among Ethiopian migrants returned from the Middle East and South Africa [16]. However, the prevalence of CMD in the current study is higher than that in the general population and working adults in the country which was ranging from 11.7% to 17.7% [19–21]. Findings of the qualitative research of this study indicated that the majority of return migrants are suffering from depression and anxiety disorders and few of them were diagnosed with a severe form of mental illness like schizophrenia. The findings of this study were in line with the study conducted in Nepal with repatriated migrants from the Gulf Arab countries and Asian countries which found that Nepalese foreign labor migrants were predominantly affected by depressive disorder and anxiety disorder [22].

The study revealed that the migrants were vulnerable to traumatic experiences and migration and adjustment related stressors. They were exposed to abuses, mistreatment, exploitation, and right violations. This was in line with a study from Sri Lanka conducted on women migrant workers returned from Middle East countries [23]. The participants in this study experienced a high level of abuse during their journey or at their stay in the Middle East countries. About 23.1%, 32%, and 55.3% of return migrants experienced sexual, physical, and verbal abuses respectively. A study conducted in Lebanon found that sexual, physical, and verbal abuses were detected in 12.5%, 37.5%, and 50.0 % of female foreign domestic workers respectively [24]. In the same way, a study from Nepal reported that 40.9% of return migrants had faced abuses at their workplace in the Middle East countries [25]. Labour migrants in the Middle East countries often encounter barriers to accessing appropriate health care [26]. In this study, only 23.2% of migrants had access to health care while they were in the Middle East countries. Similarly, a study from Nepal shows that only 12.9% of respondents reported that they had received health services after falling ill whilst in domestic work abroad [25].

Results from the multivariable analysis show that education, physical abuse, detention, salary earning, history of mental illness in the family, guilty feeling for not fulfilling expectation, and denial of access to health care were significantly associated with CMD adjusting for other possible confounding factors. In the same way, the qualitative findings identified mal-adaptation, individual susceptibility, severe abuses, painful experiences, and guilty feeling for not fulfilling the goal of the migration as causes of mental distress in Ethiopian labour migrants returned from the Middle East countries. A qualitative study done on return migrants in Ethiopia revealed that sexual violence, physical violence, emotional abuse, starvation, imprisonment, and difficulty adapting to a different culture were sources of mental trauma of Ethiopian labour migrants in the Middle East countries [17].

The study identified that only few organizations were providing rehabilitation and reintegration services for mentally ill female returnees with a very limited resource reaching a small segment of the larger population of migrant returnees [21]. Increased focus for mental health nationally and internationally, training of a relatively large number of mental health professionals, decentralization of mental health care services, support from the government, presence of St. Amanuel mental hospital, and dedication of mental health care staff are existing opportunities for the actors working on mental health care of return migrants. The major challenges of the actors identified in this study are lack of adequate funding, limited capacity to expand services, difficulty of assessing and following unaccompanied cases, low awareness, and stigma towards mental illness and high need for economic support. Some of these challenges are also reported by other studies. One study reported that mental health care actors are struggling to find consistent funding to maintain their services and only provide assistance to those in dire need [27]. Another study indicated that these organizations are overwhelmed by huge demand with limited capacity [21].

### Strength and limitations of the study

The strength of this study lies in the mixed-method design that enables it to assess the magnitude of mental distress of return migrants and different dimensions of mental health care service available to them using quantitative and qualitative approaches. The study has also a limitation. The findings of the study may not reflect the situation among all migrants returning from elsewhere as it focused only on those returned from Middle Eastern countries and during a specific time period.

## Conclusion

The prevalence of CMD is high among Ethiopian migrants who returned from the Middle East countries. Lack of pre-migration preparation and unsafe migration along with Kafala sponsorship system exposes Ethiopian labour migrants to abuses, mistreatment, exploitation, and right violations. The high proportion of Ethiopian labour migrants to the Middle East countries experienced traumatic events including physical abuse, verbal abuse, sexual violence, detention, and denial of salary.

The factors that found to be significantly associated with CMD in return migrants were education, physical abuse, salary earning, history of mental illness in the family, detention, guilty feeling for not fulfilling expectation, and denial of access to health care. Despite the high burden of mental disorder among return migrants, only a few organizations were working on mental health care targeting returnees with a very limited resource that reaching a small segment of return migrants with a mental health problem. The mental health care services were primarily targeted at and provided for female return migrants and male return migrants with mental disorders are neglected. Lack of adequate funding, limited capacity to expand services, difficulty of assessing and following up of unaccompanied patients, low awareness and stigma towards mental illness, and returnees’ high need for economic support are main challenges for the organizations working on the area.

## Data Availability

The datasets used and/or analyzed during the current study are available from the corresponding author on reasonable request.

## Abbreviations

CMD: Common mental disorder
MoLSA: Ministry of Labor and Social Affairs
SRQ-20: Self Report Questionnaire-20
UAE: United Arab Emirates
WHO: World Health Organization
